# Optical Goniometer Paired with Digital Monte Carlo Twin to Determine the Optical Properties of Turbid Media

**DOI:** 10.3390/s24113525

**Published:** 2024-05-30

**Authors:** Levin Stolz, Benedikt Beutel, Alwin Kienle, Florian Foschum

**Affiliations:** Institut für Lasertechnologien in der Medizin und Meßtechnik, Universität Ulm, Helmholtzstr 12, 89081 Ulm, Germany; benedikt.beutel@ilm-ulm.de (B.B.); alwin.kienle@ilm-ulm.de (A.K.); florian.foschum@ilm-ulm.de (F.F.)

**Keywords:** scattering anisotropy, scattering coefficient, absorption coefficient, optical parameters, Monte Carlo, goniometer, Levenberg–Marquardt, inverse problem

## Abstract

We present a goniometer designed for capturing spectral and angular-resolved data from scattering and absorbing media. The experimental apparatus is complemented by a comprehensive Monte Carlo simulation, meticulously replicating the radiative transport processes within the instrument’s optical components and simulating scattering and absorption across arbitrary volumes. Consequently, we were able to construct a precise digital replica, or “twin”, of the experimental setup. This digital counterpart enabled us to tackle the inverse problem of deducing optical parameters such as absorption and scattering coefficients, along with the scattering anisotropy factor from measurements. We achieved this by fitting Monte Carlo simulations to our goniometric measurements using a Levenberg–Marquardt algorithm. Validation of our approach was performed using polystyrene particles, characterized by Mie scattering, supplemented by a theoretical analysis of algorithmic convergence. Ultimately, we demonstrate strong agreement between optical parameters derived using our novel methodology and those obtained via established measurement protocols.

## 1. Introduction

Light propagation in turbid media plays a critical role in various fields, ranging from biomedical optics with medical applications in diagnostics and therapy [1,2,3,4,5,6] over atmospheric science [7,8] to material science in industrial applications [9]. The accurate description of light propagation relies on understanding the intricate interactions between light and scattering and absorbing particles within the medium. The radiative transport theory provides a fundamental framework for modeling such interactions, requiring precise characterization of optical properties including refractive index *n*, absorption coefficient $\mu_{a}$, scattering coefficient $\mu_{s}$ and the scattering phase function P(ϑ).

However, obtaining exact knowledge of the scattering phase function in practical applications is often challenging, as it requires precise information about the sample’s microstructure and refractive index distribution. Additionally, in numerous materials, photons frequently undergo multiple scattering events, which obscures interactions with individual scattering particles and diminish observable effects of the single scattering phase function [10,11]. Consequently, due to its simplicity, the Henyey–Greenstein approximation for the scattering phase function [12], employing the anisotropy factor *g*, has been popular in describing scattering phenomena. Given its widespread use in the field, further investigation is valuable.

Goniometers have long been instrumental in experimental measurements of scattering phase functions and optical parameters [13,14,15,16,17,18]. Despite significant advancements in experimental techniques, certain limitations persist, particularly the accuracy of comparisons between experimental data and theoretical predictions. In recent years, Monte Carlo simulations have emerged as powerful tools for describing light transport phenomena in turbid media [19,20,21]. These simulations offer the flexibility to model complex geometries and capture the effects of multiple scattering events. However, the successful implementation of Monte Carlo methods hinges heavily on precise input parameters, including the optical parameters and the accurate theoretical modeling of the experimental setup.

Addressing these challenges, we propose a novel approach that combines the strengths of goniometric measurements with Monte Carlo simulations to solve the inverse problem of finding optical parameters of an unknown turbid media. By precisely modeling our goniometric setup with a digital Monte Carlo twin, we aim to improve the accuracy and efficiency of predicting light distribution in complex scattering environments and build a quantitative method for investigating the optical properties of many materials.

## 2. Materials and Methods

### 2.1. Optical Goniometer Instrument

**Figure 1:**
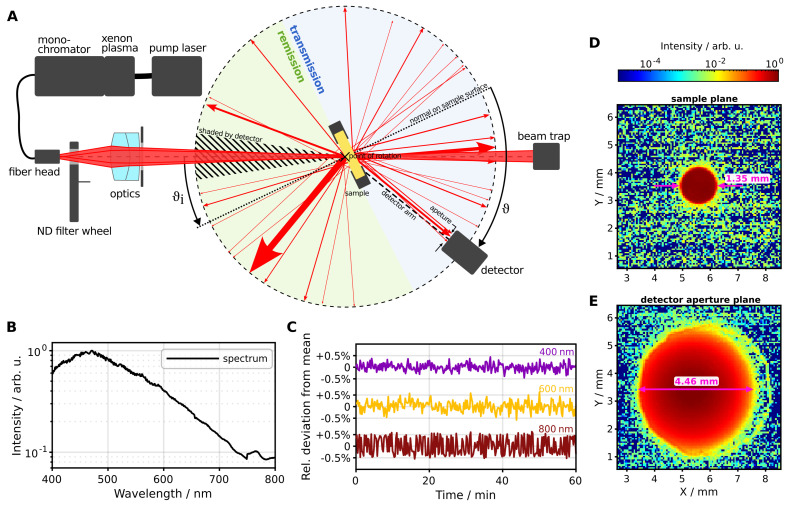
Overview of the goniometer setup. (**A**) Schematic drawing of the setup with the main optical components and angle definitions. (**B**) Emission spectrum of the light source measured with the goniometric detector. (**C**) Temporal intensity fluctuation around the mean value of the light source for three wavelengths. (**D**,**E**) Profile of the incident light in the sample plane and detector aperture plane, respectively. The images are measured with a standalone camera without any optics.

Our optical goniometer setup is depicted in Figure 1A. The setup comprises wavelength-resolved light scattering measurements at arbitrary incident and scattering angles. Both the detector and the sample are controlled by a stepper motor (Nema 11, physical resolution 1.8°, microstep resolution 0.225°) to rotate them freely through 360°, while the illumination setup remains fixed. The detector moves along a single plane around the sample, thereby limiting our ability to capture rotational asymmetric scattering patterns without manual adjustment of the sample rotation.

The illumination setup includes a laser-pumped xenon plasma source coupled directly to a single-grating monochromator (Mountain Photonics Hyperchromator II). This tunable source covers a wavelength range from 190 nm to 2500 nm with a line width of 1 nm to 10 nm full width at half maximum and measured temporal intensity stability of <±0.5% (Figure 1C). While our light source enables broad-spectrum measurements, other components within our optical system are tailored specifically to wavelengths ranging from 400 nm to 1100 nm. Consequently, we currently designate our system to operate solely within the visible to near-infrared part of the spectrum. Figure 1B displays the emission spectrum captured by the goniometric detector, showing intensity variations spanning one order of magnitude. Notably, at 750 nm, the monochromator transitions its grating, leading to a minor discontinuity in the spectrum. Following the monochromator, the light is guided through an optical multimode fiber (600 μm, NA=0.22) which distal end is located at 348 mm distance from the sample surface. Directly after the light passes through a filter wheel equipped with a pass-through port, a neutral density (ND) 1 filter and beam blocker. The filter wheel setup enables automatic dark-frame subtraction and intensity adjustment for measuring high-intensity transmission or reflection peaks as well as low-intensity scattered light within the dynamic range of the detector. Using an achromatic doublet lens (Thorlabs AC254-075-AB, f=75 mm) at 240 mm distance from the sample surface, the fiber head is imaged onto the primary sample surface, located on the rotation point of the sample stage. As sample holder, we utilize two black-coated aluminum plates with a support structure at the bottom, designed to secure the sample in place. These plates feature a 21 mm diameter cutout to enable the transmission of light.

Using an additional, standalone camera (IDS U3-3060CP-C-GL Rev.2.2) we measured the spot size of the incident beam as 1.35 mm on the sample plane (Figure 1D). Afterwards the incident beam diverges to a width of 4.46 mm at the detector aperture location (Figure 1E, 120.8 mm distance from the sample surface). A detector aperture diameter of 5 mm ensures total incident light capture at 0° during a reference measurement with an empty sample stage. With a fixed detector distance of 120.8 mm (entrance pupil), the resulting detector acceptance angle of scattered light is calculated to be 1.19°. Consequently, the angular resolution achieved is 2.38° at a detection angle of 0°, whereas it is 3.33° at a detection angle of 90° for a standard sample thickness of 2 mm (note that resolution varies slightly with changing sample thickness). This configuration offers a favorable compromise between achieving adequate angular resolution for capturing oscillations across the scattering angle and effectively integrating signal intensity to ensure low noise levels with reasonable integration times averaging 0.5 s per angle. However, our measurement abilities are constrained in a backwards direction in an area of ±8.5° around the incident beam, where the incident light is shaded by the detector.

**Figure 2:**
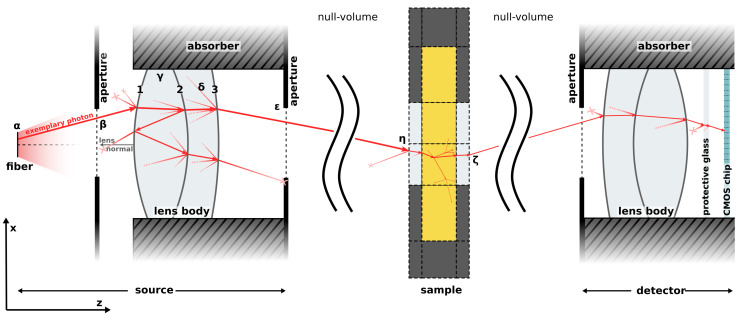
Schematic view of an exemplary photon trajectory as simulated by the Monte Carlo simulation. Dimensions are not to scale.

The detector optics include an achromatic lens (Thorlabs AC254-030-AB, f =30 mm) and a 2.3 MPixel CMOS camera (IDS U3-3060CP-M-GL Rev.2.2, 1920×1200 pixels, pixel size =5.86 μm). The precise arrangement of the detector is shown in Figure 2. During light-scattering measurements, we use a digital binning of 10×10. This optical setup enables imaging of the sample surface. Utilizing the spatial resolution capabilities of the camera chip offers the advantage of simplified alignment and, in addition to angular resolved measurements, the potential for spatially resolved measurements of the sample surface.

### 2.2. Monte Carlo Simulation of Light Transport

The Monte Carlo simulation approach to light transport involves statistically sampling the probability distributions governing propagation length and angular deflection per scattering or absorption event of a propagating photon. By simulating many photons, the resulting distribution approximates the solution of the radiative transport equation with any desired accuracy [22,23]. However, the validity of this method relies on the applicability of the Beer–Lambert law for light propagation, which assumes that the position of the scatterers is not correlated with the scattering material. In addition, each scattering and absorption event on a particle must be independent of any other particle, leading to linear absorption and scattering coefficients ($\mu_{a}$ and $\mu_{s}$) with respect to particle concentration. Additionally, due to its statistical nature, the Monte Carlo method inherently introduces statistical noise. To address this issue, modern codes, accelerated by graphics processing units (GPUs), can efficiently compute a large number of photons within a short time, often aided by variance reduction techniques [24]. Moreover, a high-quality pseudorandom number generator is essential [25] (we employed the Mersenne Twister algorithm [26]). However one of the primary advantages of Monte Carlo-based light transport simulations lies in their ability to model arbitrary geometries with diverse optical properties. In our experiment, we aim to simulate the steady-state light distribution of a goniometric measurement, considering a non-trivial sample geometry and accounting for optical effects arising from the experimental apparatus (optical fibers, lenses, apertures, sample holder, etc.).

Our simulation comprises three primary components: the source, where new photons are initiated; the sample domain, consisting of geometric volumes and associated materials with scattering and absorbing properties; and the detector regime, where photons are captured. The source and detector domains allow the arrangement of lenses and apertures in three-dimensional space. In the subsequent discussion, we picture an exemplary photon trajectory based on the configuration depicted in Figure 2. Since all optical components and scattering events in our setup are rotationally symmetric, each time a new angle is randomly drawn for the polar angle ϑ, a uniform distributed random angle between 0 and 2π is selected for the azimuthal angle φ. To accelerate our simulation massively, we leverage the parallel computing power of GPUs.

Initially, a photon is emitted from a fiber head source, initiating the photon with a uniform distribution of positions within the circular area of the fiber and with a uniform distribution of angles (ϑ and φ) within the numerical aperture (Figure 2, α). The first interaction occurs at an aperture plane (Figure 2, β). If the photon intersects outside the aperture radius, it is discarded, and the corresponding thread on the GPU starts a new photon. Intersections with a lens body (Figure 2, γ) are calculated as follows: The orientation of the lens is defined by the lens normal of interface 1. This interface the photon encounters first. The distance from the optical axis (z-axis) and the normal vector of the lens interface at the intersection point are calculated. Given the refractive indices of the bordering materials, Fresnel equations are employed to calculate the transmission or reflection probability of the photon. Optionally, the lens surface may incorporate an anti-reflection coating, in which case the probabilities for reflection and transmission are adjusted accordingly. A random number is generated to determine if the photon transmits or reflects, after which Snell’s law is applied to the incident angle ϑ. The interface number is adjusted according to the photon’s new propagation direction and it is checked whether the new interface number falls below one or exceeds the number of interfaces. If not, refraction, reflection, or transmission on the next lens interface is computed iteratively. During reflection we allow photons to be scattered on the lens surface with a small probability (Figure 2, δ). The scattering is determined by a Reynolds-McCormick phase function [27], incorporating optimized parameters $g_{s}$ and α. This approach of introducing deviations from perfection is justified in the next chapter. In the other case, the photon exits the lens body. Photons exiting in the positive direction of the lens normal of interface 1 are discarded. The final step in the source object involves determining the intersection with a second aperture plane, following the aforementioned process.

Photons departing from the source domain then enter the sample domain. This domain is described by an arrangement of cylindrical, cubic, spherical, or tetrahedral volumes. To accelerate simulation duration, only one type of volume is permitted per simulation. Each volume is characterized by its index, spatial location and size, neighboring volume indices, and material. Material properties include absorption coefficient $\mu_{a}$, scattering coefficient $\mu_{s}$, phase function P(θ), and refractive index *n*. We are able to employ various phase function models: Henyey–Greenstein, Reynolds–McCormick, double Henyey–Greenstein [28], tabular loaded from a file (e.g., calculated by Mie theory [29]) and cubic splines. Photons departing from the source at a position in 3D space and with a given direction, are intersected with the outer boundary of the sample geometry (Figure 2, η). All surface elements of the outer boundary are precalculated. At the boundary interaction, reflection or transmission is determined, and refraction is conducted according to Snell’s law. Within a volume, transmission through the volume and the propagation length of the photon is determined by sampling the probability distribution described by the Beer–Lambert law. Considering the photon’s direction, the nearest intersection point with the current volume interface is computed. If the distance is less than the current path length, transmission, reflection and refraction are computed with the neighboring volume. The photon’s position is set on the boundary. If the photon remains in the current volume after propagating the randomly chosen propagation length, absorption and scattering are determined. An absorbed photon is discarded. In the event of scattering, a scattering angle ϑ is determined by the material’s phase function, and the photon’s direction is adjusted accordingly. Should a photon transition through a boundary and enter the null volume (outside the sample; Figure 2, ζ), the last refraction is computed and it proceeds to the final step of the simulation: detection domain.

The intersection of the photon trajectory with each detector is calculated in 3D space (e.g., each angle in the goniometric simulation has its own detector). Similar to a source object, a detector may include apertures and lenses, which are computed as described previously. Additionally, refraction through a protective glass in front of the sensor chip is simulated. Lastly, the photon’s interaction with a precise pixel on the 2D detector chip is computed and stored.

### 2.3. Variance Reduction in Rotational Symmetry

**Figure 3:**
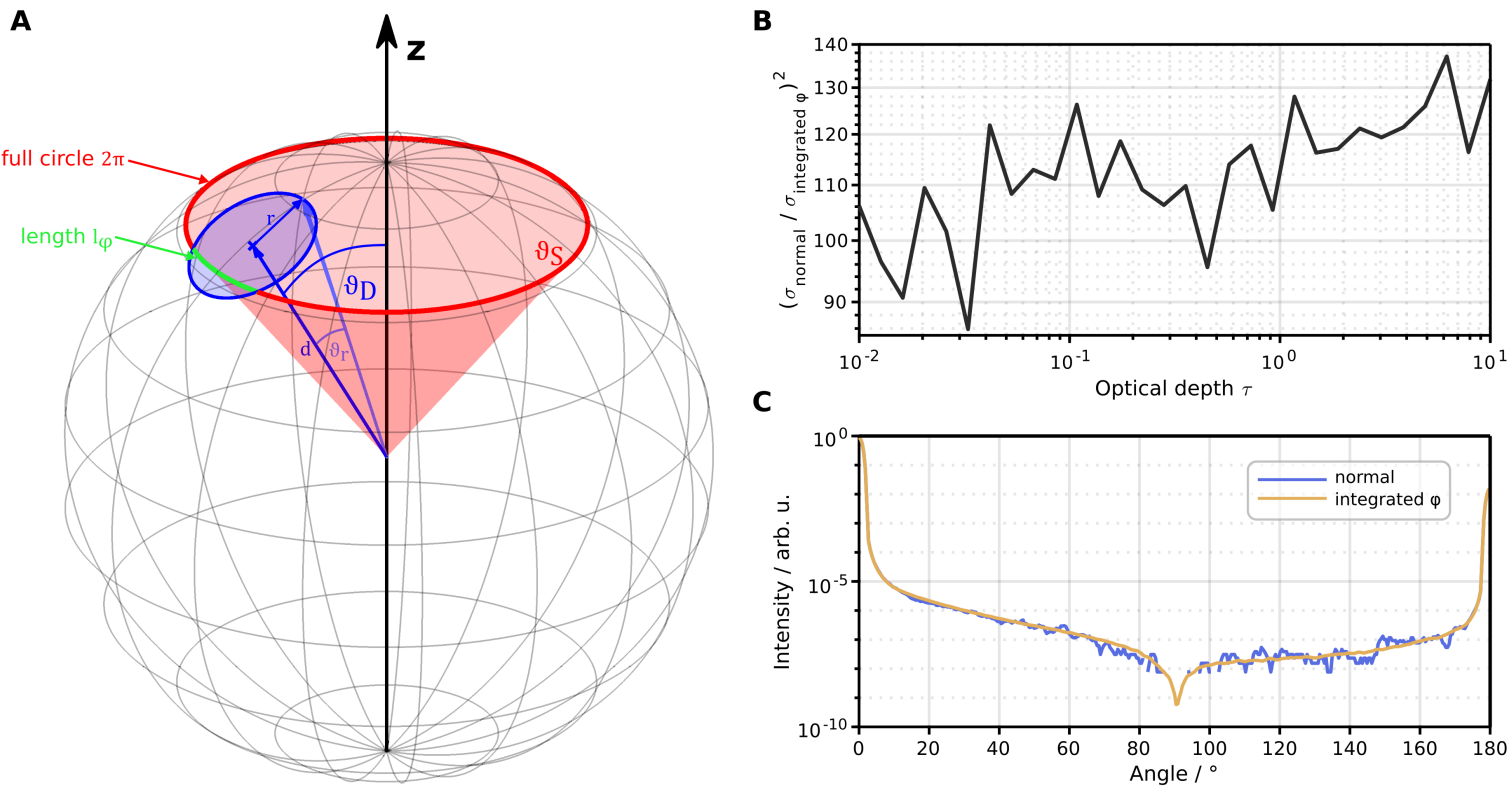
Overview of the variance reduction technique. (**A**) Schematic view of the computation problem. A photon is scattered inside the red cone given by angle $\vartheta_{S}$. A circular detector at angle $\vartheta_{D}$ detects photons along the path highlighted in green. The drawing is not to scale. (**B**) The variance reduction of integrated φ detector compared to normal, circular detector. (**C**) Comparison of the simulated goniometric signal between integrated φ and normal detector with the same simulation time. The reduction in noise is evident.

In a rotational symmetric configuration (incident angle $\theta_{i}=0$°), we can employ a variance reduction technique to speed up the Monte Carlo computation by a factor of ⪆100 (see Figure 3B) by detecting all photons with arbitrary azimuth φ coordinate, after interaction with the sample. In such instances, the simulation does not model the 2D detector chip; instead, it integrates all photons entering the detector’s aperture. However, when counting photons with arbitrary φ values, one has to be careful in calculating the normalization, since a circular detector covers a variable amount of φ-space within its extension in ϑ-space. Figure 3A shows a non-proportional view of the computation problem compared to the dimensions of our goniometer. A detector, given by a circular aperture, at angle $\vartheta_{D}$ detects photons at scattering angle $\vartheta_{S}$ at a length of $l_{\varphi }$. We can calculate this length by intersecting the cone of scattered photons $\vec{S}$ (red) with the detector cone $\vec{D}$ (blue) on the unit sphere. $\vec{S}$ is given by the scattering angle $\vartheta_{S}$ and parameter $\varphi_{S}\in \left[0,2\pi \right)$ as

(1) $$\vec{S}=\left[\begin{array}{c} \sin\vartheta_{S}\cos\varphi_{S}\\ \sin\vartheta_{S}\sin\varphi_{S}\\ \cos\vartheta_{S} \end{array}\right].$$

The detector circle $\vec{D}$ is defined by its half opening angle $\vartheta_{r}$ and parameter $\varphi_{D}\in \left[0,2\pi \right)$ and is rotated at the detector position $\vartheta_{D}$ to give

(2) $$\vec{D}=\left[\begin{array}{ccc} \cos\vartheta_{D} & 0 & \sin\vartheta_{D}\\ 0 & 1 & 0\\ -\sin\vartheta_{D} & 0 & \cos\vartheta_{D} \end{array}\right]\left[\begin{array}{c} \sin\vartheta_{r}\cos\varphi_{D}\\ \sin\vartheta_{r}\sin\varphi_{D}\\ \cos\vartheta_{r} \end{array}\right]=\left[\begin{array}{c} \cos\vartheta_{D}\sin\vartheta_{r}\cos\varphi_{D}+\sin\vartheta_{D}\cos\vartheta_{r}\\ \sin\vartheta_{r}\sin\varphi_{D}\\ -\sin\vartheta_{D}\sin\vartheta_{r}\cos\varphi_{D}+\cos\vartheta_{D}\cos\vartheta_{r} \end{array}\right].$$

The half opening angle $\vartheta_{r}$ is given by

(3) $$\tan\vartheta_{r}=\frac{r}{d},$$

where *r* is the detector radius and *d* is the distance from the origin to the detector. By intersecting $\vec{D}$ and $\vec{S}$ we obtain an expression for the φ-value of intersection as

(4) $$\vec{D}=\vec{S}\Leftrightarrow \cos\varphi_{S}=\frac{1}{\sin\vartheta_{S}}\left(\underset{=:a}{\underset{︸}{\frac{1}{\sin\vartheta_{D}\sqrt{1+\frac{r^{2}}{d^{2}}}}}}-\underset{=:b}{\underset{︸}{\frac{\cos\vartheta_{D}}{\sin\vartheta_{D}}}}\cos\vartheta_{S}\right)=:\Phi .$$

We identify *a* and *b* as detector-specific values that can be precalculated to save computation time. The weight *w* of a detected photon

(5) $$w=\frac{l_{\varphi }}{2\pi }=\frac{\arccos\Phi }{\pi }$$

is now given by the ratio of $l_{\varphi }=2\varphi_{S}$ to the full circle. If a photon is detected, its weight is calculated in the respective detector by

(6) $$w=\left\{\begin{array}{cc} \frac{\arccos\Phi }{\pi }, & \mathrm{if}\;|\Phi |<1\\ 1, & \mathrm{otherwise}. \end{array}\right.$$

### 2.4. Principle of Inverse Monte Carlo Using Parameter Perturbation

We employed our Monte Carlo simulation to solve the inverse problem of determining optical parameters that most accurately align with our goniometric measurements. The initial phase (Section 2.2) involved achieving precise reproduction of measurements through simulation, ensuring an accurate representation of the optical instrument’s impact on the data. Subsequently, we developed an efficient method for computing derivatives of the Monte Carlo simulation with respect to the fitted optical parameters (this section). These derivatives are utilized within a non-linear least squares fitting algorithm for parameter optimization. In this work, we introduce the fitting of the absorption coefficient $\mu_{a}$, the scattering coefficient $\mu_{s}$, and the anisotropy factor *g* of the Henyey–Greenstein phase function. It is noteworthy that the approach can also accommodate an arbitrary complex phase function with a larger number of parameters if needed.

We change the optical parameter *p* by a fractional value of $\delta_{p}$ as

(7) $$p^{\prime }=p\left(1+\delta_{p}\right).$$

To obtain the derivative of the Monte Carlo simulation (MC) with respect to the optical parameter *p*,

(8) $$\frac{\partial \mathrm{MC}}{\partial p}=\frac{\mathrm{MC}\left(p^{\prime }\right)-\mathrm{MC}\left(p\right)}{p^{\prime }-p},$$

we use the finite difference method. To calculate the derivative of *N* optical parameters one could perform N+1 Monte Carlo simulations (one base simulation of *p* and *N* simulations of each varied parameter $p^{\prime }$). However, as already widely used in Monte Carlo simulations, we can make use of perturbation Monte Carlo (pMC) to efficiently compute small perturbations $\delta_{p}$ of a background Monte Carlo simulation [30,31,32,33,34]. In pMC, the simulation is scaled by a scaling function $S_{p}$ to change the contribution of the parameter *p* as

(9) $$\mathrm{MC}\left(p^{\prime }\right)=S_{p}\left(\mathrm{MC}\left(p\right)\right).$$

Inserting Equations (7) and (9) into Equation (8) gives the derivative of the Monte Carlo simulation

(10) $$\frac{\partial \mathrm{MC}}{\partial p}=\frac{S_{p}\left(\mathrm{MC}\left(p\right)\right)-\mathrm{MC}\left(p\right)}{p\delta_{p}},$$

with respect to *p* from a single simulation. We can apply $S_{p}$ by scaling the contribution of each photon path to the total intensity with a photon weight $w_{p}$. For each optical parameter $\mu_{a}$, $\mu_{s}$, and *g*, we can find an expression for its weight $w_{p}$ when perturbed by a factor $\left(1+\delta_{p}\right)$.

By varying the absorption coefficient, we modify the likelihood of the photon transmission. The transmission probability *T* is described by the Beer-Lambert law. The weight of a photon for a scaled absorption results from the ratio of transmission probabilities and is expressed as

(11) $$\begin{array}{cc} w_{\mu_{a}} & =\frac{T_{\mu_{a}^{\prime }}}{T_{\mu_{a}}}, \end{array}$$

(12) $$\begin{array}{cc} \; & =\frac{\exp\left(-l\mu_{a}\left(1+\delta_{\mu_{a}}\right)\right)}{\exp\left(-l\mu_{a}\right)}, \end{array}$$

(13) $$\begin{array}{cc} \; & =\exp\left(-l\delta_{\mu_{a}}\mu_{a}\right), \end{array}$$

where *l* is the path length of the photon inside the absorbing medium.

For scaling the scattering coefficient $\mu_{s}$, one has to consider the change in transmission as well as the change of scattering probability. The transmission probability is calculated analogously to $w_{\mu_{a}}$. The scattering probability of a single scattering event changes by

(14) $$\frac{\mu_{s}^{\prime }}{\mu_{s}}=1+\delta_{\mu_{s}}.$$

Combined with $N_{scat}$ as the number of scattering events for each photon and the transmission probability following Beer-Lambert law, we obtain

(15) $$w_{\mu_{s}}={\left(1+\delta_{\mu_{s}}\right)}^{N_{scat}}\exp\left(-l\delta_{\mu_{s}}\mu_{s}\right),$$

for the scaled $\mu_{s}$ photon weight.

To scale the Henyey–Greenstein phase function parameter *g*, one must multiply the probability change of scattering at angle $\vartheta_{s}$ for each scattering event $i=1,\ldots ,N_{\mathrm{scat}}$ as

(16) $$w_{g}=\prod_{i=1}^{N_{scat}}\frac{P{\left(\vartheta_{s,i}\right)}_{g^{\prime }}}{P{\left(\vartheta_{s,i}\right)}_{g}}.$$

Using Equations (10), (13), (15) and (16) enables us to receive not only the simulated goniometric signal but also its derivative with respect to the optical parameters $\mu_{a}$, $\mu_{s}$, and *g* from a single Monte Carlo simulation.

Lastly, we want to minimize

(17) $$C=\frac{\log\mathrm{MC}-\log E}{\log E},$$

where *C* is the cost function and *E* is our measured experimental goniometric signal. We apply a logarithmic transformation to our signals due to the wide range of intensity values observed in goniometric measurements, spanning multiple orders of magnitude for ballistic and scattered light. This approach secures the numerical stability of our fitting algorithm. The chain rule must be employed to compute the derivatives of the cost function *C*,

(18) $$\frac{\partial C}{\partial p}=\frac{\partial C}{\partial \mathrm{MC}}\frac{\partial \mathrm{MC}}{\partial p}=\frac{1}{\mathrm{MC}\log E}\frac{\partial \mathrm{MC}}{\partial p},$$

with respect to the optical parameters *p*.

To solve the non-linear problem of minimizing *C*, we apply the Levenberg–Marquardt algorithm using the derivatives ∂C/∂p to calculate the Jacobian matrix. Typically we fit around 100 measured angles in one nonlinear regression.

### 2.5. Bounds Implementation in Levenberg–Marquardt Algorithm

In this section, we discuss the incorporation of boundary constraints into the Levenberg–Marquardt algorithm for parameter fitting. Specifically, for optical parameters, we confine the absorption and scattering coefficients to the range of zero to infinity (unrestricted), and the g-factor to the range of minus one to one (or equivalently, from zero to one, as negative values for the g-factor are typically not observed in practical scenarios). Simply imposing hard limits on these parameters can disrupt the algorithm’s ability to compute derivatives when the parameters fall outside the constrained interval, leading to unstable outcomes.

To address this, we utilized a parameter transformation between the bounded parameter space, denoted as $P_{B}$, and the internal (unbounded) parameter space, denoted as $P_{I}$. Here, the bounded parameters are employed by the Monte Carlo simulation, while the internal parameters are used by the Levenberg–Marquardt optimization algorithm. The transformation functions are taken from the MINUIT2 software library [35]. However, since the Monte Carlo simulation computes derivatives with respect to the bounded parameter space ($\partial \mathrm{MC}/\partial P_{B}$), while we aim to optimize the internal parameter space, an additional chain rule is introduced in Equation (18) as

(19) $$\frac{\partial \mathrm{MC}}{\partial p}=\frac{\partial \mathrm{MC}}{\partial P_{I}}=\frac{\partial \mathrm{MC}}{\partial P_{B}}\frac{\partial P_{B}}{\partial P_{I}}.$$

In the following, we state the equations for the parameter transformations, taken from MINUIT2, and give the scaling factor $\partial P_{B}/\partial P_{I}$, considering scenarios where parameters are solely lower-bounded between *a* and infinity, solely upper-bounded between minus infinity and *b*, and completely bounded within the range of *a* to *b*. Lower bounded:

(20) $$\begin{array}{cc} P_{I} & =\sqrt{{\left(P_{B}-a+1\right)}^{2}-1}, \end{array}$$

(21) $$\begin{array}{cc} P_{B} & =a-1+\sqrt{P_{I}^{2}+1}, \end{array}$$

(22) $$\begin{array}{cc} \frac{\partial P_{B}}{\partial P_{I}} & =\frac{P_{I}}{\sqrt{P_{I}^{2}+1}}. \end{array}$$

Upper bounded:

(23) $$\begin{array}{cc} P_{I} & =\sqrt{{\left(b-P_{B}+1\right)}^{2}-1}, \end{array}$$

(24) $$\begin{array}{cc} P_{B} & =b+1-\sqrt{P_{I}^{2}+1}, \end{array}$$

(25) $$\begin{array}{cc} \frac{\partial P_{B}}{\partial P_{I}} & =\frac{-P_{I}}{\sqrt{P_{I}^{2}+1}}. \end{array}$$

Completely bounded:

(26) $$\begin{array}{cc} P_{I} & =\arcsin\left(\frac{2(P_{B}-a)}{b-a}-1\right), \end{array}$$

(27) $$\begin{array}{cc} P_{B} & =b+\left(\sin\left(P_{I}\right)+1\right)\frac{b-a}{2}, \end{array}$$

(28) $$\begin{array}{cc} \frac{\partial P_{B}}{\partial P_{I}} & =\frac{b-a}{2}\cos\left(P_{I}\right). \end{array}$$

## 3. Results

### 3.1. Validation Measurements

We performed validation measurements to test the precision of the Monte Carlo simulation in reproducing experimental results. In both simulation and experiment, the entire 2D detector chip is integrated for each position of the goniometric arm. Normalization of each angular measurement is carried out based on its integration time, with adjustments made for any wavelength-dependent attenuation of the incident beam if an ND filter was utilized during irradiation. To enable comparison between measurement and simulation, calibration values are applied to each by simulating, respectively, measuring the incident radiation under 0° with no sample present (in the Monte Carlo simulation, this corresponds to a thin layer of air being simulated).

**Figure 4:**
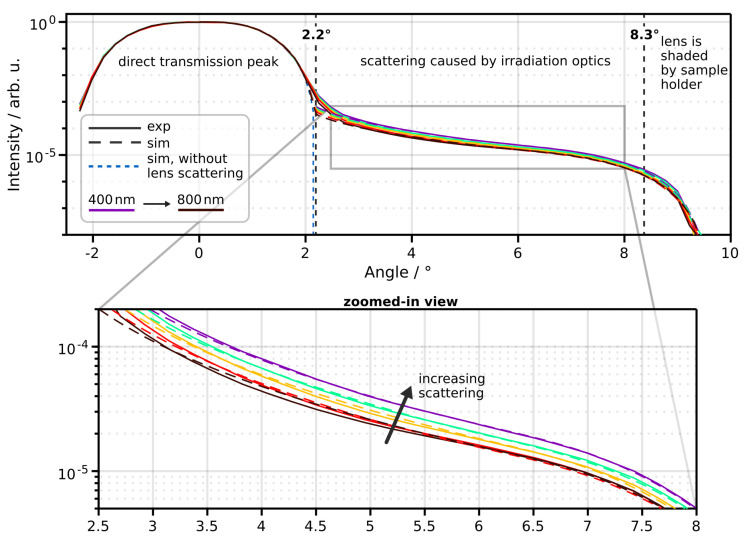
Comparison between measured and simulated instrument response function for five wavelengths. The tailing signal at angles >2.2° is attributed to lens surface scattering as it increases with decreasing wavelength (zoomed-in view).

In an initial test, goniometric measurements were conducted without a sample to validate our irradiation modeling. Figure 4 shows the instrument response function (IRF) across five wavelengths. Within the range of zero to two degrees, the direct transmission peak is observed. The profile of the transmission peak is primarily shaped by the optical characteristics of the optical fiber (including diameter and numerical aperture) and lenses (such as refractive indices and surface curvature). With our relatively precise knowledge of these parameters (obtained from the manufacturer), simulation accuracy in this regime is exceptionally high.

Angles exceeding two degrees reveal an underlying signal attributed to scattering caused by the irradiation optics. This assertion is corroborated by the observed increase in the amplitude of the underlying signal with decreasing wavelength (zoomed-in view Figure 4). In our simulation, we modeled the scattering phenomenon with a Reynolds-McCormick phase function parameterized by $g_{s}$ and α. Additionally, we applied a scattering probability factor. The specific wavelength-dependent values were determined through manual iterations of Monte Carlo simulations for each wavelength. The simulated signal without this introduced scattering effect is shown as a dashed blue line in Figure 4. Notably, the signal decrease starting at around 8° is due to the extended sample holder (with a cutout of 21 mm in diameter) shadowing the irradiation optics. The comparison between the experiment and simulation shows good agreement with our employed model.

**Figure 5:**
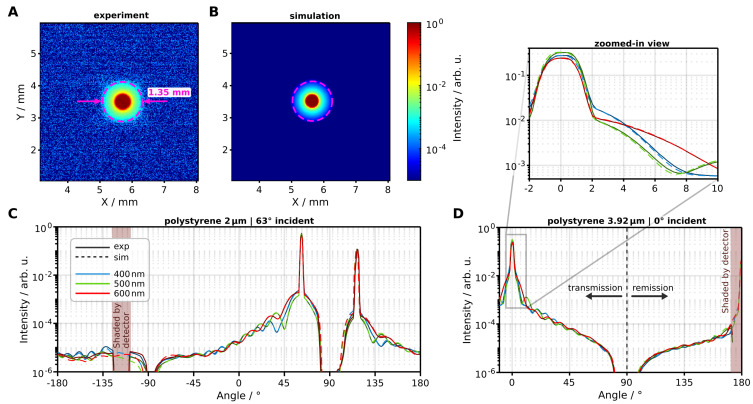
Overview of the geometric validation measurements. (**A**,**B**) View of the measured and simulated 2D detector image at 0° incident light and empty sample stage. The spot size of 1.35 mm corresponds to the imaged spot size on the sample surface. In these images, no binning of the detector pixels was applied. (**C**,**D**) Comparison at three wavelengths between measured and simulated goniometric signal for polystyrene spheres with 2 μm (**C**) and 3.97 μm (**D**) in diameter.

Figure 5A,B show a comparison between experimental and simulated detector images for an empty sample stage. We measured a spot size of 1.35 mm which corresponds to the imaged spot size on the sample front plane (see Figure 1D). In the simulated image, we observe a marginally steeper decrease in the background signal around the irritation peak, although with minimal absolute deviation. We attribute this deviation to our modeling of lens imperfections as surface scattering in the simulation. Nonetheless, while our model does not precisely mirror the spatial signal distribution on the detector chip, it characterizes with high agreement the integrated signal behavior in angular resolved measurements (refer to Figure 4).

In the subsequent phase, we investigated a theoretical verifiable model system. The probing samples used in these measurements necessitate precise characterization of their optical properties. To fulfill this requirement, we utilized spherical polystyrene microparticles. Such particles are known for their uniform shape and are available in various diameters from commercial suppliers. Since the diameter, size distribution, and refractive index of these spherical particles are relatively well known, we were able to employ Mie theory to compute the theoretical scattering phase function P(θ) and scattering coefficient $\mu_{s}$. Data for the refractive index of polystyrene were taken from [36]. We measured polystyrene particles with diameter 2.0 μm and 3.92 μm (microParticles GmbH, Berlin, Germany, concentration given by the manufacturer =0.1%) suspended in water. With further dilution, we obtained the values presented in Table 1. We simulated the polystyrene samples with zero absorption.

To measure liquid samples, we used a custom-made glass cuvette composed of two circular N-BK7 glass panels (each with a thickness of 1.035 mm) and a precise aluminum spacer ring. In our simulations, the cuvette is modeled as two glass volumes separated by an intermediate scattering volume, enclosed within a surrounding sample holder. The glass volumes are characterized by zero absorption and scattering. The refractive index of N-BK7 glass is sourced from literature references [37]. Additionally, we estimated the refractive index of the scattering medium as that of water. This choice is based on the effective medium approximation, which suggests that at low concentrations of scattering inclusions in a matrix material, the effective refractive index tends to align with the refractive index of the matrix medium itself [38]. In our simulations, we adjusted the particle diameter of the larger particles from 3.97 μm (as given by the manufacturer) to 3.92 μm to align more closely with the experimental measurements. This adjusted size falls within the standard deviation and is supported by collimated transmission measurements conducted by Pink et al. on the same sample [39]. Experiment and simulation data were normalized following the procedures outlined previously.

Our validation measurements show very good agreement between goniometric measurement and Monte Carlo simulation. This is especially true for the shape of the transmission peak, where the influence of the optical setup like lenses and apertures are most prominent in the signal.

### 3.2. Convergence of Inverse Monte Carlo Tested on Forward Simulation

**Figure 6:**
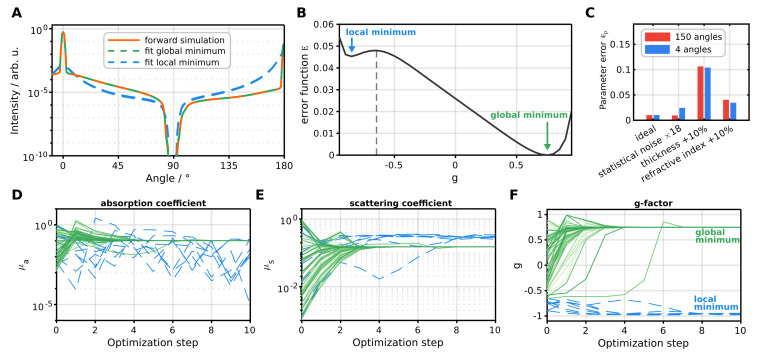
Convergence analysis of the inverse algorithm on forward simulation. (**A**) Comparison between forward simulation and fitted signals. The local minimum deviates noticeably from the optimum solution. (**B**) Error function E=∥C∥ as a function of the g-factor. A local and global minimum are separated by an extremum value at g=−0.65. (**C**) Parameter error, calculated by Equation (29), for various error scenarios and different amounts of fitted angles. (**D**–**F**) Fitted optical parameters as a function of optimization steps. The start values (step 0) are chosen randomly.

We tested the performance of our inverse algorithm using a Monte Carlo forward simulation. In the forward simulation, we modeled a glass cuvette with an intermediate scattering medium. The medium possesses optical properties characterized by $\mu_{a}=0.1{\mathrm{mm}}^{-1}$, $\mu_{s}=0.15{\mathrm{mm}}^{-1}$, g=0.75, $n_{\mathrm{med}}=1.33$, and a thickness of $t_{\mathrm{scat}}=2$ mm. The glass, with a refractive index of $n_{\mathrm{glass}}=1.51$, has a thickness of $t_{\mathrm{glass}}=1\mathrm{mm}$. The simulated ideal forward calculation includes statistical simulation noise of 0.25%, averaged over all angles, and is depicted in orange in Figure 6A.

We fitted 150 angles from 0° to 170°, excluding the interval between 80° and 100°. Figure 6B shows the error function E=∥C∥, derived from the cost function *C* (Equation (17)), across varying *g* values and ideal absorption and scattering coefficients. We see the emergence of a global and local minimum at g=0.75 and g=−0.85, respectively, and an extremum at g=−0.65. Our convergence analysis involved conducting 80 fits with all randomly initialized optical parameters. Trajectories in Figure 6D–F depict the evolution of fit parameters through optimization steps. Green trajectories converge to the global minimum, while blue trajectories converge to the local minimum. Notably, convergence towards the local minimum occurs when the initial *g*-factor value is below the extremum, irrespective of the starting absorption and scattering coefficients. This convergence towards the local minimum results in a notably incorrect fit (Figure 6A, blue), with a fit error reaching 350% (as computed by Equation (30)). The local minimum represents a solution where the fit parameters reach a locally optimal configuration. However, it may not necessarily correspond to the global optimal solution and may exhibit low accuracy with a large discrepancy between fitted and forward simulated signals. Conversely, starting parameters that converge towards the global minimum demonstrate excellent accuracy (fit error of 0.34%, as depicted by the green curve in Figure 6A), with most cases achieving rapid convergence within five optimization steps.

In our implementation of the inverse algorithm, we restricted the g-factor to positive values only, as non-positive values are not encountered in practical applications [1,3,8]. Although this approach eliminates the local minima encountered in this scenario, the possibility of additional local minima in other fitting scenarios cannot be disregarded. To address the issue of local minima, we conduct fitting with multiple different initial parameters when the initial fit fails to achieve satisfactory minimization of the fit error.

To evaluate the convergence of the inverse algorithm, we calculated the

(29) $$\begin{array}{cc} \mathrm{parameter}\;\mathrm{error}\;\epsilon_{p} & =\frac{1}{3}\sum_{p=\mu_{a},\mu_{s},g}\left|\frac{p_{\mathrm{fit}}-p_{\mathrm{forward}}}{p_{\mathrm{forward}}}\right|, \end{array}$$

(30) $$\begin{array}{cc} \mathrm{fit}\;\mathrm{error}\;\epsilon_{\mathrm{fit}} & =\sqrt{\frac{1}{N_{\mathrm{angles}}}\sum_{i=1}^{N_{\mathrm{angles}}}{\left(\frac{{\mathrm{MC}}_{\mathrm{fit}}\left(i\right)-{\mathrm{MC}}_{\mathrm{forward}}\left(i\right)}{{\mathrm{MC}}_{\mathrm{forward}}\left(i\right)}\right)}^{2}}, \end{array}$$

and

(31) $$\begin{array}{cc} \mathrm{simulation}\;\mathrm{noise}\;\sigma & =\sqrt{\frac{1}{N_{\mathrm{angles}}}\sum_{i=1}^{N_{\mathrm{angles}}}\left(\frac{1}{N_{\mathrm{photons},\mathrm{forward}}\left(i\right)}+\frac{1}{N_{\mathrm{photons},\mathrm{fit}}\left(i\right)}\right)}. \end{array}$$

Figure 6C shows the parameter errors for 150 and 4 fitted angles, respectively, obtained from the ideal forward simulation (leftmost) and three adjusted forward simulations representing potential error scenarios. We introduced a statistical noise increase by a factor of 18 (from 0.25% (ideal forward simulation) to 4.5%), achieved by simulating a factor of 324 fewer photons. For 150 fitted angles, we observe no significant alteration in the accuracy of the inverse algorithm; however, the relative error between forward simulation and fitted signal averaged over all fitted angles increases from 0.34% to 3.3%. This can be attributed to the overconstrained nature of the model, where the 150 fit angles act as a form of averaging. With a reduced number of fit angles, we achieve similar convergence as with all 150 angles in the low-noise ideal case. However, in the noisy case, we observe an increase in parameter error since no averaging takes place. Introducing systematic errors in the fitting process, such as increasing the sample thickness or the refractive index of the scattering medium by 10%, results in parameter errors of ≈11% and ≈4%, respectively. When fitting a faulty thickness, the resultant fit error remains relatively small at 1.3% (for 150 angles) and 0.0032% (for 4 angles), making it more challenging to detect the systematic error compared to a faulty refractive index, which leads to an increased error of 4.6% (for 150 angles) and 0.027% (for 4 angles).

**Figure 7:**
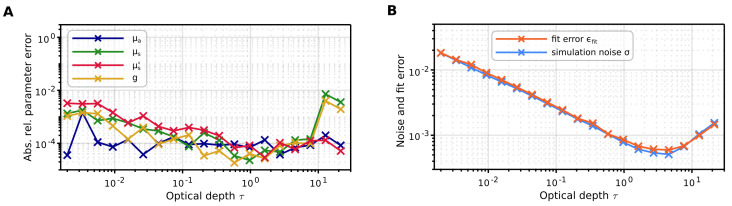
Convergence of the inverse algorithm for increasing optical depth $\tau =t_{\mathrm{scat}}\mu_{s}$. (**A**) Absolute relative parameter error $\left|\left(p_{\mathrm{fit}}-p_{\mathrm{forward}}\right)/p_{\mathrm{forward}}\right|$. (**B**) Fit error and simulation noise calculated by Equations (30) and (31), respectively.

Finally, we assessed the performance of the inverse algorithm as the optical depth increased. We conducted forward simulations of a single layer with fixed parameters: $\mu_{a}=0.1{\mathrm{mm}}^{-1}$, g=0.65, $n_{\mathrm{med}}=1$, $t_{\mathrm{scat}}=2\mathrm{mm}$, and varied the scattering coefficient $\mu_{s}$ from $0.001{\mathrm{mm}}^{-1}$ to $10{\mathrm{mm}}^{-1}$. The inverse Monte Carlo algorithm was utilized to fit the optical parameters $\mu_{a}$, $\mu_{s}$, and *g*, from which the reduced scattering coefficient $\mu_{s}^{\prime }=\mu_{s}\left(1-g\right)$ was derived. Figure 7B shows the optimal convergence achieved in the fitting process for all optical depths, as the fit error aligns with the statistical noise of the simulation. We obtained satisfactory errors in the fitted optical parameters of <0.5% for optical depths ≤10, beyond which the determination of $\mu_{s}$ and *g* deteriorates. Nonetheless, the determination of $\mu_{a}$ and the combined parameter $\mu_{s}^{\prime }$ remains viable even for large optical depths.

### 3.3. Fitting Monte Carlo Simulations to Goniometric Measurements

We put to use our inverse algorithm on goniometric measurements to test the performance against an established measurement routine. We employed an in-house-produced optical phantom composed of a silicon matrix material, zirconium oxide scatter particles (ZrO, diameter 800 nm, concentration 0.057%), and iron oxide pigment (mixture of FeO(OH) and Fe_2_O_3_, concentration 0.003%). The refractive index of the silicon matrix material was measured with an ellipsometer by Wagner et al. [40]. For obtaining reference data, we measured the optical parameters $\mu_{a}$, $\mu_{s}$, and *g* using two setups: a thick sample ($t_{\mathrm{scat}}=6\mathrm{mm}$ and $t_{\mathrm{scat}}=9\mathrm{mm}$) was applied for an integrating sphere setup to determine $\mu_{a}$ and $\mu_{s}^{\prime }$ [41], and a thin sample ($t_{\mathrm{scat}}=3\mathrm{mm}$) for a collimated transmission setup to determine $\mu_{t}$ [39]. From these measurements, we calculated the remaining quantities

(32) $$\begin{array}{cc} \mu_{s} & =\mu_{t}-\mu_{a}, \end{array}$$

(33) $$\begin{array}{cc} g & =1-\frac{\mu_{s}^{\prime }}{\mu_{s}}. \end{array}$$

**Figure 8:**
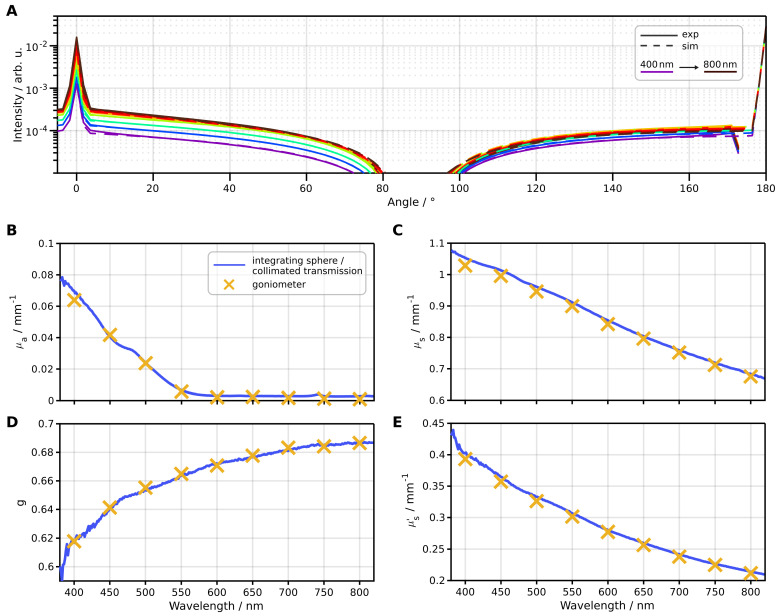
Geometric measurements evaluated with the presented inverse algorithm and compared to combined integrating sphere and collimated transmission measurements. (**A**) Comparison of the measured and fitted goniometric signal. (**B**–**E**) Comparison of the optical parameters $\mu_{a}$, $\mu_{s}$, *g* and $\mu_{s}^{\prime }$, solved from goniometric measurements (yellow crosses) and determined from other measurements (blue line).

Using the goniometer setup, we measured the 3 mm thick sample at nine wavelengths ranging from 400 nm to 800 nm, with 73 angles fitted for each wavelength. We performed eight optimization steps per wavelength. We fitted the parameters $\mu_{a}$, $\mu_{s}$ and *g* and calculate the parameter $\mu_{s}^{\prime }$. The fitting process for all nine wavelengths required approximately 11 min on a single NVIDIA GeForce 4070 Ti GPU. As presented in Figure 8A, the goniometric measurements and the corresponding Monte Carlo simulations show a strong agreement. The fitted optical parameters, illustrated in Figure 8B–D, exhibit mean relative differences between the goniometric and reference measurements of 3.9%, 1.4%, 0.17% and 1.5% for $\mu_{a}$, $\mu_{s}$, *g* and $\mu_{s}^{\prime }$, respectively, for absolute values absorption values greater than $0.01{\mathrm{mm}}^{-1}$. It is noteworthy that the accuracy of determining the absorption coefficient diminishes for absolute values below $0.01{\mathrm{mm}}^{-1}$. At this threshold, we anticipate a relative error of the absorption coefficient of 17% based on the measured source fluctuation of 0.5%.

## 4. Discussion

In this study, we demonstrated a high level of agreement between our optical goniometer and its digital counterpart, generated through Monte Carlo simulations. This is achieved through meticulous consideration of all relevant components contributing to the measurement signal. Furthermore, our apparatus, along with its digital twin, enables reliable optical parameter determination of volume scattering materials extending up to an optical depth of 10, as shown for the case of volume scattering determined by a Henyey–Greenstein phase function. From our goniometric measurements, we successfully extracted the absorption and scattering coefficients, as well as the anisotropy factor, which were further validated against established measurement protocols. When analyzing scattering samples using a combination of integrating sphere and collimated transmission measurements, it becomes necessary to employ both thick (high optical depth) and thin (low optical depth) samples. With our novel inverse goniometric approach, we can extract optical information even from samples where providing optical depths over multiple scales is not possible.

Nevertheless, it is essential to acknowledge that many materials, especially those in the low scattering regime, may not be adequately characterized solely by the Henyey–Greenstein phase function. In our upcoming publication, we will extend the investigations into the challenges encountered when employing the Henyey-Greenstein function, particularly when single scattering events are influenced by more complex phase functions. Nonetheless, our developed inverse algorithm offers versatility, as it allows for the incorporation of alternative fitted phase functions such as Raynolds–McCormick and those constructed using cubic splines.

## Figures and Tables

**Table 1:** Measured validation samples consisting of suspended polystyrene particles in water. The scattering coefficient ${\bar{\mu }}_{s}$ is averaged over the three analyzed wavelengths.

Particle Diameter	Standard Deviation	Concentration Solids Content	${\bar{\mu }}_{s}$	Sample Thickness
2 μm	<0.03 mm	0.0097%	0.16 mm^−1^	2.08 mm
3.92 μm	<0.06 mm	0.037%	0.32 mm^−1^	3.93 mm

## Data Availability

The data that support the findings of this study are available from the corresponding author, L.S., upon reasonable request.
